# Pseudo-squared: Pseudomonas pseudo-outbreak lab investigation

**DOI:** 10.1017/ash.2025.10220

**Published:** 2025-12-10

**Authors:** Shinya Hasegawa, Poorani Sekar, Bradley Ford, Katie Halliwill, Karen Brust

**Affiliations:** 1 Division of Infectious Diseases, Department of Internal Medicine, University of Iowa Carver College of Medicine, Iowa City, IA, USA; 2 Department of Pathology, University of Iowa Carver College of Medicine, Iowa City, IA, USA; 3 Program of Hospital Epidemiology, Quality Improvement Program, University of Iowa Carver College of Medicine, Iowa City, IA, USA

Distinguishing outbreaks from pseudo-outbreaks is essential in healthcare settings. Pseudo-outbreaks are defined by an increase in identified organisms without clinical evidence of infection. Here we report two cases involving *Pseudomonas aeruginosa* identified in clinical specimens, later determined to represent a pseudo-outbreak.

Patient #1 had vertebral osteomyelitis and epidural abscess; intraoperative and blood cultures grew a bacterium in the *Streptococcus mitis/oralis* group. Four days postsurgery, one colony of *P. aeruginosa* grew from one of three intraoperative aerobic cultures. Patient #2 developed a fracture-related infection of the ankle and underwent debridement and hardware removal; all intraoperative cultures grew methicillin-susceptible *Staphylococcus aureus*. Four days later, two colonies of *P. aeruginosa* were detected in one of three intraoperative aerobic cultures. Both these findings were deemed unusual by the clinician seeing both patients, leading to an outbreak investigation, including chart review and laboratory investigations, to identify a source of contamination.

**Figure 1.**
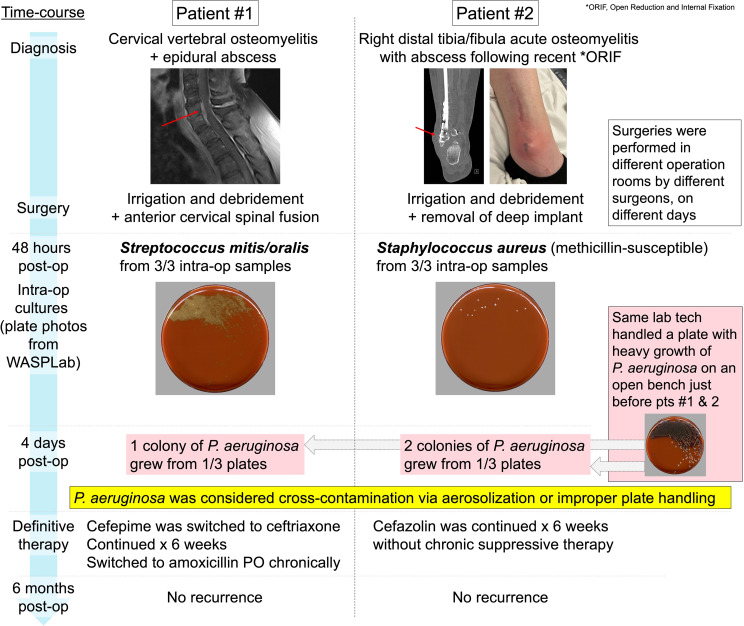
Timeline of the outbreak investigation and the patient’s clinical course.

The two cultures were received and set up one day apart by different staff on the WASPLab (Walk-Away Specimen Processor system),^1^ which is an automated system for incubation, culture processing and preserving plate images. It has been reported to be effective for reducing incubation times and allowing early culture readings^2,3^ because it is a closed system, which in principle also reduces points of contamination. The WASPLab incubation system’s photographic record of the plates demonstrated no *P. aeruginosa* within the expected first 48 hours, suggesting that contamination during culture collection or processing was unlikely. Further investigation revealed that a plate with heavy growth of *P. aeruginosa* was processed by the same laboratory technician on an open bench immediately before handling plates for patients #1 and #2. *P. aeruginosa* typically grows rapidly, and the colony morphology of the contaminated plates matched those of the culture with heavy growth. Furthermore, both patients had monomicrobial growth of a likely pathogen causing their infection. Therefore, we concluded that this was cross-contamination, likely via aerosolization during plate handling. For patient #1, cefepime was discontinued on postoperative day six and switched to ceftriaxone, completed for six weeks, followed by suppressive therapy with amoxicillin, with no recurrence at six months. Patient #2 completed six weeks of cefazolin, also without recurrence at six months (Figure 1).

We identified a pseudo-outbreak of *Pseudomonas aeruginosa*, which led to narrowing of antibiotic therapy and improved antimicrobial stewardship without recurrence at follow-up. Several key findings supported this being a pseudo-outbreak rather than true infection. First, *P. aeruginosa* typically grows rapidly under standard laboratory conditions, making delayed or unexpected colony appearance suspicious. Second, the colony morphology on the implicated plates matched that of a heavily inoculated source culture, suggesting a common origin. Third, both affected patients had monomicrobial cultures of organisms typically associated with clinical infection, reducing the likelihood of true polymicrobial infection involving *P. aeruginosa*.

The most plausible mechanism of contamination was aerosolization during processing at an open bench. Although aerosolization is not often documented as a source of plate contamination, the circumstantial evidence in this case was compelling. The fact that heavily inoculated *P. aeruginosa* cultures were handled in close proximity and immediately before the affected specimens on an open bench at the time of processing further support this hypothesis.

This event underscores several key aspects of hospital epidemiology. First, laboratory procedures must minimize the risk of cross-contamination, particularly when handling high-burden or hazardous organisms. Aerosolization risks, while uncommon, should be appreciated, especially with pathogens such as *Brucella* spp., known for causing laboratory-acquired infections.^4^ Second, digital imaging of culture plates and meticulous laboratory documentation can be instrumental for outbreak investigations. In this case, serial images from the WASPLab system helped determine the timing of growth and exclude early contamination. Third, clinical-microbiological correlation is essential. Over-reliance on unexpected culture results can lead to inappropriate treatment. Collaboration among microbiologists, infectious diseases providers, and hospital epidemioloogists leads to accurate interpretation, avoidance of unnecessary antimicrobial exposure, improved stewardship outcomes, and favorable clinical outcomes. Recently, whole-genome sequencing (WGS) has emerged as a reliable tool for nosocomial outbreak surveillance.^5, 6^ Performing WGS in this case could have further confirmed our theory.

In conclusion, this investigation highlights the importance of distinguishing pseudo-outbreaks from true infections. Cross-contamination during laboratory processing, though rare, can lead to diagnostic confusion and unnecessary antimicrobial exposure. Digital tools, laboratory vigilance, and multidisciplinary clinical correlation are critical for accurate diagnosis and effective stewardship.
